# Evaluating the accuracy and speed of eight deduplication tools: A comparative study

**DOI:** 10.1017/rsm.2026.10100

**Published:** 2026-06-17

**Authors:** Sarah Bateup, Helen Fulbright, Klas Moberg, Kaitlyn Hair, Emmy Peterson, Claire M. Stansfield, Riaz Qureshi, Justin Clark

**Affiliations:** 1 Library Services, Bond University, Gold Coast, Australia; 2 Centre for Reviews and Dissemination, University of York, UK; 3 Swedish Agency for Health Technology Assessment and Assessment of Social Services, Sweden; 4 EPPI Centre, UCL Social Research Institute, University College London, UK; 5 Department of Ophthalmology, School of Medicine, University of Colorado, Anschutz Medical Campus, USA; 6 Institute for Evidence-Based Healthcare, Bond University, Gold Coast, Australia

**Keywords:** automation, evidence synthesis, systematic reviews, deduplication, systematic review software, duplicate references

## Abstract

A key task in conducting systematic reviews is deduplicating the results from database searching. Deduplication using reference management software can be time-consuming and prone to error, while automated tools can be expensive and lack transparency. To support review teams, we evaluated eight deduplication tools: (1) The Automated Systematic Search Deduplicator (ASySD); (2) Covidence; (3) Deduklick; (4) EPPI-Reviewer; (5) PICO Portal; (6) Rayyan; (7) The Systematic Review Accelerator (SRA) Deduplicator: Focused; (8) The SRA Deduplicator: Relaxed. Five randomly selected Cochrane reviews had their searches rerun to create five gold standard sets. We compared the gold standard sets to the outputs of the eight deduplication tools and evaluated the results for: (1) unique records removed; (2) duplicate records retained; (3) time taken to deduplicate. Summed across all five reviews, the unique records removed in error ranged from 2 to 22. The three best tools were: (1) Rayyan; (2) Covidence; (3) SRA Deduplicator: Focused. The duplicate records retained in error ranged from 34 to 280, the three best tools were: (1) ASySD; (2) Rayyan; (3) EPPI-Reviewer. The time taken to deduplicate ranged from one minute to 20 hours and 34 minutes, the three fastest tools were: (1) SRA Deduplicator: Relaxed; (2) Deduklick; (3) Covidence. No tool performed so poorly that we don’t recommend using it. But, as all the tools had strengths and weaknesses, some are expensive while others require large amounts of manual checking time, we recommend review teams compare the tools across all three outcomes and choose the tool that best suits their needs.

## Highlights

### What is already known?

- A key task in conducting a systematic review is to find all relevant studies, often through searching multiple databases.
- An overlap in database journal coverage produces duplicate records, and these must be removed before screening in a process known as ‘deduplication’.
- Various automation tools are available to assist with deduplication.

### What is new?

- This comparative study includes a bigger range of automation tools than other studies.
- The methods are more robust, with two authors checking the results of each tool, comparing their findings, and resolving conflicts.
- This study includes the time spent using each automation tool.

### Potential impact for *RSM* readers

- The findings of this study are informative to help evidence synthesis teams compare the performance of eight deduplication tools, across the important outcomes of (1) singulars removed, (2) duplicate records retained, and (3) time spent using the tool for deduplication .

## 1 Introduction

Systematic reviews (SRs) require a substantial investment of time and resources taking, on average, 67 weeks to complete and costing USD $141,000.^1^ ^,^ ^2^ Systematic review automation (SRA) tools have been developed to help reduce this substantial resource burden. These SRA tools aim to improve the speed of SRs without impacting their quality.^3^

A key task when conducting a SR is to find all relevant studies, and this is typically achieved by searching multiple databases.^4^ As there is overlap in the journals indexed in each database, searches usually return large numbers of duplicate records. These duplicate records should be removed for efficiency before the results of the searches can be assessed for relevance. This removal of duplicate records is normally referred to as deduplication.

The deduplication of search results can be performed in numerous ways.^5^

Historically, reference management software (RMS), such as EndNote, Zotero, or Mendeley have been used to compile records from all sources (i.e., databases), although the use of SR tools for deduplication is increasing. Using RMS, deduplication is achieved using a semi-manual method that combines the RMS’s deduplication component, with a review of the results by humans.^6^ This method is time-consuming and, even with humans reviewing the results, prone to errors. An alternative way is using automation tools.^7^ ^,^ ^8^ These tools can bring their own problems, the first being their accessibility through expensive proprietary software. The second is that some operate in a ‘black box’ environment, meaning that humans cannot check the results or properly understand how these were obtained. The third remaining problem is that no one is sure how these tools compare with each other and which tool is best suited for defined purposes.

To address these issues, we undertook a comparative study of eight deduplication tools with the objective of providing evidence to help information specialists or review teams determine which tool best suits their needs.

## 2 Methods

This study evaluates the performance of eight deduplication tools in their deduplication of search results from five randomly selected Cochrane reviews. The study was designed using the automatic protocol writing tool available in the Systematic Review Accelerator (SRA) called the Trial Wizard.^9^

To obtain the sample sets for deduplication, we randomly identified five recent Cochrane reviews and reran their searches. The search results were then deduplicated using the following eight tools: (1) The Automated Systematic Search Deduplicator (ASySD)^10^; (2) Covidence^11^; (3) Deduklick^12^; (4) EPPI-Reviewer^13^; (5) PICO Portal^14^; (6) Rayyan^15^; (7) The SRA Deduplicator: Focused^16^; (8) The SRA Deduplicator: Relaxed.^16^ We compared the tools across the following three outcomes: (1) singulars (unique studies) removed (false positives); (2) duplicate records retained (false negatives); (3) time spent using the tool for deduplication.

A summary of the methodology is as follows, we:

1. Selected five Cochrane reviews.
2. Obtained the sample to be deduplicated.
3. Created the gold standard sets.
4. Tested each of the eight tools against the gold standard sets.
5. Compared each tool’s performance against the three stated outcomes.

### 2.1 Selection of five Cochrane reviews

To ensure an unbiased sample of search results, we randomly selected five Cochrane reviews published within a two-year period (Jan 2020–Dec 2022). We chose to use Cochrane reviews as these are required to search two or more databases and report their search strings for all databases. The reviews were randomly selected by running the following search string in PubMed:

- ((‘Cochrane Database Syst Rev’[jour]) AND (2020/01/01:2022/12/31[Date - Publication]))

The search results were then ordered from newest to oldest using the ‘Most recent’ display option in PubMed. Next, we used Google’s random number generator to generate a number between one and the total number of search results found (1,469). The PubMed search result that corresponded to the random number was checked to ensure it met our inclusion criteria. We performed this process until five eligible Cochrane reviews had been identified.

To be eligible for inclusion in our study, the search strategies of Cochrane reviews had to meet the following criteria:

- The review was completed and published.
- Search strings for all bibliographic databases searched were reported.
- The number of bibliographic databases searched was between three and eight.
- The total number of search results found by the combination of all database searches was between 1,000 and 20,000 records.

The decision to limit the number of databases used and the total number of search results was to reduce variability between samples and to ensure they were representative of typical SRs, which search five databases and retrieve a median of 1,781 search results after duplicates are removed.^2^

### 2.2 Obtaining the sample to be deduplicated

After the five eligible Cochrane reviews were selected, the sample to be deduplicated was obtained by rerunning all the bibliographic database search strings that had been used within the review. This led to the creation of five separate datasets.

For a search to be rerun, the appropriate database needed to be accessible and usable by the study team. In some cases, the study team had access to a database but via a different platform. For example, if Embase was originally searched via Ovid, but the team has access to Embase via Elsevier, the search string was translated either manually or via the Polyglot Search Translator^17^ and then run. No date or language limits were applied, even if they were applied in the original Cochrane review. Searches of specialised registers, trial registries, or grey literature sources listed in the Cochrane reviews were excluded as we did not have access to specialised registers, and the formats output by trial registries or grey literature searches were generally not usable by the deduplication tools at the time of the study. The samples were created during Spring 2023.

### 2.3 Creating the gold standard sets

Two study authors (J.C. and S.B.), who are experienced information specialists, independently deduplicated the search results from each of the five datasets. This was done by uploading the references to EndNote, manually sorting the references by multiple fields (e.g., title, then author etc.), and removing any duplicates found. They then compared their results and resolved disputes through discussion. This created five ‘gold standard’ sets of search results to be used in the study.

In creating our gold standard sets and throughout this study, we defined a singular as an article published in a unique place, that is, unique volume, issue, and page range within a journal, or the same article published in different places. A good example of the latter is publications related to the preferred reporting items for systematic reviews and meta-analyses (PRISMA) statement, which were published in multiple journals. We defined a duplicate as the same article published within the same volume and issue of a journal.

*These are singulars*:

Preferred reporting items for systematic reviews and meta-analyses: the PRISMA statement. Moher D, Liberati A, Tetzlaff J, Altman DG; PRISMA Group. *Int J Surg*. 2010;8(5):336–41. doi:10.1016/j.ijsu.2010.02.007.

Preferred reporting items for systematic reviews and meta-analyses: the PRISMA statement. Moher D, Liberati A, Tetzlaff J, Altman DG; PRISMA Group. *J Clin Epidemiol*. 2009 Oct;62(10):1006–12. doi:10.1016/j.jclinepi.2009.06.005.

Preferred reporting items for systematic reviews and meta-analyses: the PRISMA statement. Moher D, Liberati A, Tetzlaff J, Altman DG; PRISMA Group. *BMJ*. 2009 Jul 21;339:b2535. doi:10.1136/bmj.b2535.

*These are duplicates*:

Preferred reporting items for systematic reviews and meta-analyses: the PRISMA statement. Moher D, Liberati A, Tetzlaff J, Altman DG; PRISMA Group. *J Clin Epidemiol*. 2009 Oct;62(10):1006–12. doi:10.1016/j.jclinepi.2009 .06.005

Preferred reporting items for systematic reviews and meta-analyses: The PRISMA statement. Moher, D., Liberati, A., Tetzlaff, J., Altman, D. G. (2009). *Journal of Clinical Epidemiology*, 62(10), 1006–1012. 10.1016/j.jclinepi.2009.06.005

Moher D, Liberati A, Tetzlaff J, Altman DG. Preferred reporting items for systematic reviews and meta-analyses: the PRISMA statement. *J Clin Epidemiol*. 2009;62(10):1006–1012. doi:10.1016/j.jclinepi.2009.06.005

### 2.4. Testing the eight deduplication tools

We ran the five datasets in each of the deduplication tools: ASySD, Covidence, Deduklick, EPPI-Reviewer, PICO Portal, Rayyan, SRA Deduplicator: Focused, and SRA Deduplicator: Relaxed. This was performed by various members of the study team. Due to differences in the design of the tools, some of the deduplication was fully automated (Deduklick, SRA Deduplicator: Relaxed, Covidence), whereas other tools (EPPI-Reviewer, Rayyan, SRA Deduplicator: Focused, PICO Portal, ASySD) were processed semi-automatically which also required some manual decision-making by the tester. EPPI-Reviewer’s automated deduplicated feature was set at 0.85 (followed by manual checking). The Rayyan Systematic Auto-Resolver (available in the subscription version) was set to auto-delete all duplicates with a similarity rate of 100% leaving a larger amount of potential duplicates to manual decision-making; deduplication criteria for specific fields were not applied. PICO Portal deduplication automates with high certainty (e.g., identical reference information) and triggers manual review for potential matches when there is lower confidence, such as when there are fewer than three matching metadata fields and the title or author does not match, or the title similarity is above 0.8 but the abstract similarity is below 0.7. For this study, the Deduplicator was used on the SRA platform, however, the Deduplicator is now available at the Evidence Review Accelerator (TERA, https://tera-tools.com/). The Deduplicator in TERA is the same as it was on the SRA platform. During the testing process, each tester noted the time required to deduplicate the dataset, inclusive of automation and manual verification.

After deduplication had been completed, we exported the deduplicated set from the tool into EndNote 21 to compare the results to the gold standard sets. To minimise bias, two members of the study team independently examined the EndNote libraries for each sample and marked records that met the following outcomes:

- Singulars removed (false positives): the number of records in the sample set that the tool classified as a duplicate when it was a unique record. Singular removal is a negative outcome, and having a low number is a high priority for deduplication because classification of unique, singular records as duplicates deletes them from the pool of eligible records, meaning that relevant records may be erroneously excluded.
- Missed duplicates (false negatives): the number of records in the sample set that the tool classified as a unique record when it was a duplicate record. Missed duplicates are also a negative outcome for deduplication tools; however it is less important than the incorrect removal of singulars as a missed duplicate can be excluded during screening by the review team.

The third outcome was recorded by the participants while using each of the tools.

- Time required to deduplicate: the time required to deduplicate the sample. Time is an important consideration and includes the full process (i.e., automated deduplication plus any additional manual checking that is required).

The removed singulars and missed duplicates were recorded in an Excel spreadsheet or Word document to compare and resolve disagreements through discussion. Where agreement could not be reached, the decision was made by a third member of the study team (J.C.).

### 2.5 Comparison of outcomes

Upon completion of the study, the three outcomes measured were recorded for each tool for each sample set.

#### 2.5.1 Statistical methods

A linear mixed model was fitted to compare the time taken, and logistic mixed models were fitted to compare the proportion of errors (separate models for missed duplicates and removed singulars) by the eight tools used for deduplication. A random intercept was specified to account for within review correlation and least-squares means were estimated and compared across the eight tools. Time taken was log-transformed prior to analysis to remove positive skew. Statistical Analysis System (SAS) OnDemand for Academics was used for the statistical analysis.

Statistical ranking of the methods was not performed for missing singulars due to insufficient numbers of errors to allow this ranking to be accurately performed. All model outputs, including the Poisson model, have been included in the Supplementary Material.

## 3 Results

### 3.1 Characteristics of the deduplication tools evaluated

There were eight deduplication tools evaluated as part of this study. The SRA has two deduplication tools, both of which were evaluated. The tools ranged from those that are fully automated (3 of 8) to those partially automated with some human checking (5 of 8). We evaluated both subscription-based tools (5 of 8) and free tools (3 of 8) (Table 1).

**Table 1:** Characteristics of the tools evaluated Table 1 long description.

Tool name	Date of evaluation	Cost	Degree of automation
ASySD	February 2025	Free	Partially automated, some manual checking required
Covidence	March 2024	Subscription	Fully automated
Deduklick	February/March 2024	Subscription	Fully automatic
EPPI-Reviewer	September 2024	Subscription	Partially automated, some manual checking required
PICO Portal	February 2025	Subscription	Partially automated, some manual checking required
Rayyan	December 2023	Subscription	Partially automated, some manual checking required
SRA Deduplicator: Focused	October 2024	Free	Partially automated, some manual checking required
SRA Deduplicator: Relaxed	July 2024	Free	Fully automated

### 3.2 Characteristics of the reviews used in the evaluation (gold standard)

We deduplicated the search results of five reviews for this study. All five reviews included randomised controlled trials (RCTs), quasi-RCTs, or controlled clinical trials. Their publication years ranged from 2020 to 2022. Three databases were searched for four out of the five Cochrane reviews, with the remaining review searching four databases. The number of search results to be deduplicated ranged from 2,524 to 12,432, while the number of duplicates removed ranged from 397 to 5,145 (Table 2). The percentage of duplicates within each dataset varied from just 8% to 58.6%. The total number of duplicates removed across all five reviews was 7,247.

**Table 2:** Characteristics of the Cochrane reviews used in the evaluation Table 2 long description.

Dataset	Title	Types of studies included in review	Databases from original review included in deduplication study (platforms accessed in the original reviews)	Number of records with duplicates	Duplicates removed	Number of records in deduplicated set (gold standard)
Arora 2022	School dental screening programmes for oral health	RCTs; cluster- or individually randomised	MEDLINE (Ovid), Embase (Ovid), CENTRAL (Cochrane Library)	3,299	533	2,766
Cox 2021	Telerehabilitation for chronic respiratory disease	RCTs and controlled clinical trials	MEDLINE (Ovid), Embase (Ovid), CENTRAL (Cochrane Library)	5,258	397	4,861
Freak-Poli 2020	Workplace pedometer interventions for increasing physical activity	RCTs	CENTRAL (Cochrane Library), CINAHL (EBSCO), MEDLINE (Ovid), Embase (Elsevier)	12,432	5,145	7,287
Noone 2020	Video calls for reducing social isolation and loneliness in older people: a rapid review	RCTs and quasi-RCTs (including cluster designs)	MEDLINE (Ovid), CINAHL (EBSCO), PsycInfo (Ovid)	3,265	397	2,868
Pisano 2021	Renal denervation for resistant hypertension	RCTs	MEDLINE (Ovid), Embase (Ovid), CENTRAL (Cochrane Library)	2,524	775	1,749

### 3.3 Overall performance of deduplication tools

Of the 22,778 records spanning all five reviews, the tools identified and removed between 6,976 and 7,248 duplicate records (Table 3). As the tools varied in performance across each of the outcomes measured in this study, no single tool was best across each outcome. The most important criteria for many review teams will be the lowest number of missed singulars, as it is highly favourable not to miss potentially relevant studies. For this outcome, the best performing tool was Rayyan. Of 22,778 records across all five reviews, Rayyan removed only two singulars overall, whereas ASySD performed worst, with 22 singulars removed. For duplicate identification, ASySD performed best, with only 34 duplicates missed (an overall sensitivity of 99.5%), while the worst performer was SRA Deduplicator: Relaxed, with 280 duplicates missed (overall sensitivity of 98.6%). In terms of the time taken, the SRA Deduplicator: Relaxed was the fastest, taking 42 seconds in total to deduplicate all five libraries, whereas Rayyan was the slowest at 20 hours and 34 minutes in total (Table 3).

**Table 3:** Performance of individual tools for deduplication across all outcomes Table 3 long description.

Tool	Total records with duplicates	Duplicates removed	Records in deduplicated sets	Removed singulars	Missed duplicates	Total time (HH:MM)
ASySD	26,778	7,248	19,530	22	34	00:53
Covidence	26,778	6,985	19,793	8	276	00:32
Deduklick	26,778	7,189	19,589	11	81	00:14
EPPI-Reviewer	26,778	7,209	19,569	20	74	01:03
PICO Portal	26,778	7,129	19,649	16	136	05:07
Rayyan	26,778	7,222	19,556	2	40	20:34
SRA Deduplicator: Focused	26,778	7,191	19,587	9	81	02:58
SRA Deduplicator: Relaxed	26,778	6,976	19,802	11	280	00:01

### 3.4 Results of statistical methods

Descriptive statistics on the variables available for analysis are shown below, stratified by the eight deduplication tools. Means for all outcomes for all tools, with both the standard deviation (SD) and the ranges are listed by tool (Table 4).

**Table 4:** Means, standard deviations (SD) and ranges of outcomes Table 4 long description.

Tool	Total records with duplicates (SD): range	Records in deduplicated set (SD): range	Removed singulars (SD)	Missed duplicates (SD): range	Mean time (SD): range minutes
ASySD	5,356 (4,084): 2,524 to 12,432	3,906 (2,207): 1,744 to 7,302	4 (3): 0 to 7	7 (10): 1 to 25	11 (7): 6 to 23
Covidence	5,357 (4,084): 2,524 to 12,432	3,959 (2,251): 1,786 to 7,450	2 (1): 0 to 3	55 (64): 21 to 169	7 (5): 4 to 14
Deduklick	5,358 (4,084): 2,524 to 12,432	3,918 (2,217): 1,753 to 7,333	2 (1): 1 to 4	16 (23): 5 to 57	3 (1): 1 to 5
EPPI-Reviewer	5,359 (4,084): 2,524 to 12,432	3,914 (2,208): 1,758 to 7,316	4 (3) 1 to 8	15 (17): 3 to 44	13 (8): 3 to 24
PICO Portal	5,360 (4,084): 2,524 to 12,432	3,930 (2,225): 1,778 to 7,372	3 (2) 1 to 6	27 (36): 5 to 90	61 (80): 11 to 201
Rayyan	5,361 (4,084): 2,524 to 12,432	3,911 (2,205): 1,755 to 7,305	0 (1) 0 to 1	8 (10): 3 to 25	247 (292): 79 to 766
SRA Deduplicator: Focused	5,363 (4,084): 2,524 to 12,432	3,917 (2,211) 1,753 to 7,323	2 (1): 0 to 4	16 (18) 2 to 45	36 (27): 8 to 80
SRA Deduplicator: Relaxed	5,362 (4,084): 2,524 to 12432	3,960 (2,258): 1,779 to 7,464	2 (1): 1 to 4	56 (68) 22 to 177	0.1 (0.1): 0.08 to 0.32

*Note:* SD, standard deviation.

### 3.5 Removed singulars

Logistic mixed model analysis of errors due to removed singulars found some evidence of a difference between the deduplication tools (p = 0.020), and comparison of least-squares means identified that the difference was due to Rayyan having significantly less errors than the other tools, except Covidence and SRA Deduplicator: Focused. We also found that Covidence had a lower error rate than ASySD and EPPI-Reviewer. The SRA Deduplicator: Focused had a lower error rate than ASySD. Below is a table of the least-squares mean error rate for each of the eight deduplication tools. Statistical ranking of the tools is not presented due to insufficient numbers of errors to allow this ranking to be accurately performed (Table 5).

**Table 5:** Removed singulars Table 5 long description.

Tool	Estimate	Standard error	Mean error rate	Standard error mean error rate
Rayyan	–9.4049	0.7520	0.000082	0.000062
Covidence	–8.0183	0.4365	0.000329	0.000144
SRA Deduplicator: Focused	–7.9005	0.4202	0.000370	0.000156
Deduklick	–7.6997	0.3955	0.000453	0.000179
SRA Deduplicator: Relaxed	–7.6997	0.3955	0.000453	0.000179
PICO Portal	–7.3248	0.3578	0.000659	0.000235
EPPI-Reviewer	–7.1014	0.3398	0.000823	0.000280
ASySD	–7.0060	0.3331	0.000906	0.000301

### 3.6 Missed duplicates

Logistic mixed model analysis of errors due to missed duplicates found very strong evidence of a difference between the deduplication tools (p < 0.0001) and comparison of least-squares means identified that ASySD and Rayyan had the lowest error rates, followed by Deduklick, EPPI-Reviewer, and SRA Deduplicator: Focused, and then PICO Portal. The most error-prone tools were Covidence and SRA Deduplicator: Relaxed. Below is a table of the least-squares mean error rate for each of the eight deduplication tools, ranked by error rate and statistical ranking; note that equal statistical rankings mean there was no statistical difference between the tools (Table 6).

**Table 6:** Missed duplicates Table 6 long description.

Tool	Estimate	Standard error	Mean error rate	Statistical ranking	Standard error mean error rate
ASySD	–6.8921	0.3063	0.001015	1	0.000311
Rayyan	–6.7293	0.2990	0.001194	1	0.000357
EPPI-Reviewer	–6.1127	0.2792	0.002210	2	0.000616
Deduklick	–6.0220	0.2771	0.002419	2	0.000669
SRA Deduplicator: Focused	–6.0220	0.2771	0.002419	2	0.000669
PICO Portal	–5.5014	0.2679	0.004065	3	0.001084
Covidence	–4.7875	0.2608	0.008265	4	0.002138
SRA Deduplicator: Relaxed	–4.7729	0.2607	0.008385	4	0.002168

### 3.7 Time to deduplicate

Linear mixed model analysis of log time taken found very strong evidence of a difference between the deduplication tools (p < 0.0001), and comparison of least-squares means identified that SRA Deduplicator: Relaxed was faster than all the other tools, Deduklick was next fastest; followed by Covidence; then ASySD; then EPPI-Reviewer; then PICO Portal; then SRA Deduplicator: Focused and finally Rayyan, which was significantly slower than all the other tools. These times included both the automatic identification and any manual verification of duplicates, where relevant, which was a substantial portion of the time. Below is a table of the least-squares mean time taken in minutes for each of the eight deduplication tools ranked by error rate and statistical ranking; note that equal statistical rankings mean there was no statistical difference between the tools (Table 7).

**Table 7:** Time to deduplicate Table 7 long description.

Tool	Estimate	Standard error	Mean time in minutes	Statistical ranking
SRA Deduplicator: Relaxed	–2.1239	0.3489	0.11	1
Deduklick	0.9000	0.3489	2.4	2
Covidence	1.6809	0.3489	5.3	3
ASySD	2.2095	0.3489	9.1	3
EPPI Reviewer	2.3406	0.3489	10.3	3
SRA Deduplicator: Focused	3.3330	0.3489	28.0	4
PICO Portal	3.5127	0.3489	33.5	4
Rayyan	5.1022	0.3489	164.3	5

## 4 Discussion

This study evaluated eight deduplication tools across three outcomes: removed singulars, missed duplicates, and time to deduplicate. The results show each tool had strengths and limitations. None made significant mistakes when removing singulars, which is the most important consideration when deduplicating. Some missed more duplicates than others, meaning an increase in screening burden as they must be identified during screening. Some tools are subscription-based, while others are free. Additionally, some require time invested in manual screening, while others are fully automated with no manual checking. There is no clear ‘best’ tool across the three outcomes measured.

There are other studies which have looked at various tools for deduplicating records.^8^ ^,^ ^18^ ^–^ ^20^ One study,^8^ published in 2021, evaluated Ovid multifile search, EndNote desktop, Mendeley, Zotero, Covidence, and Rayyan, only two of which are automation tools: Covidence and Rayyan. They deduplicated only a single set of search results (3,130 records) and found similar results to ours for Covidence (2 removed singulars, 120 missed duplicates), but their Rayyan results varied substantially (52 removed singulars, 49 missed duplicates). This was probably caused by relying overly on the automatic deduplication process within Rayyan, where we used substantial manual review.

A follow-up to this study, published in 2024, added the following to their original results: EndNote 20, EndNote Online Classic, ProQuest RefWorks, Deduklick, and the SRA Deduplicator, although they did not note whether they used the Relaxed or Focused tools.^20^ Their results for Deduklick (2 removed singulars, 109 missed duplicates) and the SRA Deduplicator (11 removed singulars, 35 missed duplicates) were different from ours.

A third study,^19^ conducted in 2022, again only deduplicated a single library of 5,933 records and evaluated EndNote X9, Mendeley, Zotero, Rayyan, and the SRA Deduplicator; again no mention of whether Relaxed or Focused was used. They found both Rayyan and the SRA Deduplicator accurate enough to be used to deduplicate records.

The fourth study,^18^ published in 2024, used six sets of search results from SRs ranging from 300 to 1,000 records. They evaluated Covidence, Deduklick, the SRA Deduplicator: Focused, the SRA Deduplicator: Relaxed, and Rayyan. Their results were similar to ours, with all tools only removing a few singular records overall and successfully identifying most of the duplicates. They also measured time and again had similar results with the fully automated tools completing the duplication much faster than the tools that require manual checking.

Our robust study design has evaluated and compared a much larger number of tools than other studies. We are the first to use two independent people to create the gold standard, assess the results of each tool, and compare and talk through any disagreements. This gives us the most accurate results to date. With the growth of generative artificial intelligence (GenAI), there are new automation tools being released onto the market. Future studies could focus on evaluating these new tools, which could be compared to our results.

The significant variability across the study outcomes has an impact on how the tools should be used. As some tools (e.g., EPPI-Reviewer and Rayyan) offer a range of thresholds for the automatic detection of duplicates, this impacts their performance across all outcomes measured. Moreover, as some tools involved manually reviewing the results (e.g., EPPI-Reviewer and Rayyan), this also affects the outcome of time taken to deduplicate. The variability in the tools, regarding results, speed, and pricing also makes it difficult for us to recommend one over any of the others. All tools performed similarly in their removal of singulars, which is the most important outcome: if a tool had performed poorly for this outcome, we would not recommend it. Therefore, information specialists and the review team should select the tool that best fits the needs of their review, whether this is the accuracy of the tools, their budget, or amount of time available. Well-funded research teams could choose the subscription tools, while those teams on a more limited budget could choose the free tools.

### 4.1 Strength and limitations

Our study has numerous strengths. The first is that we randomly identified the record sets to be used in the study, opposed to using a convenience sample. The second is that we used search results from five SRs, rather than focusing on only one or two reviews. The third, and probably most impactful, is that we had the gold standard reference set and the results from each tool checked by two people independently, who compared their results. This led to an extremely accurate set of results for each of the outcomes.

Our study also has weaknesses. The first was the fact that different people with different skills and experience did the manual checks and comparisons for the tools. This variation in skills and experience means other people could get different results when they use the tools. Having different pairs of study team members check and compare their findings means that the results across tools are not as comparable as they would have been if the same person did the manual reviewing for each tool. The second is that, despite our best efforts, the gold standard sets contained a small number of errors. As errors were identified late in the study, it was not feasible to retrospectively rerun the analysis of the tools. But as all tools were compared to the same gold standard sets, they were all evaluated in the same way, which reduces the impact this would have on the study results. The third is that all the sets of records deduplicated came from health databases, minimising the applicability of the results to non-health reviews. Fourth, we did not characterise the types of errors produced by each deduplication tool. For example, the removal of a unique study due to the same research being published in multiple journals (whether legally or otherwise) is likely to have less real-world impact than the removal of a study that closely resembles another in bibliographic information, for example, a near-identical title ending in ‘part I’ versus ‘part II’. Finally, we did not formally assess the transparency or usability of the deduplication tools. Some tools provide access to their source code, which enhances understanding for users with coding expertise. Others offer options for manual verification of identified duplicates or provide detailed written documentation on the logic and bibliographic fields used to make deduplication decisions. Future work could evaluate these aspects to help reviewers select the most appropriate tool for different review teams and circumstances, while also providing guidance to tool developers on key considerations.

Although this study only considers certain healthcare databases, which may limit the applicability to databases in other domains, it includes a bigger range of automation tools than other studies and adds to the paucity of literature in this area.

All five SRs analysed in this study included well-defined study types, such as RCTs, quasi-RCTs, or controlled clinical trials. All five reviews included in this study employed validated search filters for trials that are not necessarily available for other quantitative and qualitative SR questions. Reviews that include less well-defined study types, for example, cohort studies or qualitative evidence, may identify more duplicates. This means that the results of the deduplication tools may be different for less well-defined study types.

The small sample size for this study means that our statistical results should be interpreted with caution.

## 5 Conclusion

We evaluated eight tools and could determine that there was no single tool that performed better than the others on all three outcomes measured. All tools performed to a high enough standard for us to recommend them, so users should select the one that best fits their needs, for example, the amount of time and funds they have available for the deduplication task when synthesising evidence. Further studies could evaluate new tools or the results from trial registry searches, grey literature searches, non-health review searches, searches from alternate platforms, or in different languages.

Overall, the findings of this study are informative to help evidence synthesis teams compare the performance of eight deduplication tools across the important outcomes of (1) singulars removed, (2) duplicate records retained, and 3) time spent using the tool for deduplication.

## Supporting information

10.1017/rsm.2026.10100.sm001 Bateup et al. supplementary material Bateup et al. supplementary material

## Data Availability

The data that support the findings of this study are derived from published systematic reviews, which were accessed through institutional database subscriptions. The data used as the comparison to evaluate the tools (the gold standard sets) are available through the Open Science Framework: https://osf.io/xu3tj

## Supplementary material

To view supplementary material for this article, please visit http://doi.org/10.1017/rsm.2026.10100.

## Long descriptions

### Table 1 Long description

The header row lists Tool name, Date of evaluation, Cost, and Degree of automation. The first row is A S y S D, evaluated February 2025, free, partially automated with some manual checking required. The second row is Covidence, evaluated March 2024, subscription, fully automated. The third row is Deduklick, evaluated February or March 2024, subscription, fully automatic. The fourth row is EPPI dash Reviewer, evaluated September 2024, subscription, partially automated with some manual checking required. The fifth row is PICO Portal, evaluated February 2025, subscription, partially automated with some manual checking required. The sixth row is Rayyan, evaluated December 2023, subscription, partially automated with some manual checking required. The seventh row is S R A Deduplicator Focused, evaluated October 2024, free, partially automated with some manual checking required. The eighth row is S R A Deduplicator Relaxed, evaluated July 2024, free, fully automated. The tools differ in degree of automation, with some fully automated and others requiring manual checking. Cost varies between free and subscription. Evaluation dates range from December 2023 to February 2025.

Navigate back to Table 1.

### Table 2 Long description

From top to bottom, the table lists five datasets: Arora 2022, Cox 2021, Freak-Poli 2020, Noone 2020, and Pisano 2021. For Arora 2022, the title is School dental screening programmes for oral health, study types are R C Ts, cluster- or individually randomised, databases are MEDLINE Ovid, Embase Ovid, and CENTRAL Cochrane Library, with 3,299 records with duplicates, 533 duplicates removed, and 2,766 in the deduplicated set. Cox 2021 covers Telerehabilitation for chronic respiratory disease, includes R C Ts and controlled clinical trials, uses MEDLINE Ovid, Embase Ovid, and CENTRAL Cochrane Library, with 5,258 records with duplicates, 397 duplicates removed, and 4,861 in the deduplicated set. Freak-Poli 2020 addresses Workplace pedometer interventions for increasing physical activity, includes R C Ts, uses CENTRAL Cochrane Library, CINAHLEBS CO, MEDLINE Ovid, and Embase Elsevier, with 12,432 records with duplicates, 5,145 duplicates removed, and 7,287 in the deduplicated set. Noone 2020 is about Video calls for reducing social isolation and loneliness in older people, includes R C Ts and quasi-R C Ts including cluster designs, uses MEDLINE Ovid, CINAHLEBS CO, and PsycInfo Ovid, with 3,265 records with duplicates, 397 duplicates removed, and 2,868 in the deduplicated set. Pisano 2021 covers Renal denervation for resistant hypertension, includes R C Ts, uses MEDLINE Ovid, Embase Ovid, and CENTRAL Cochrane Library, with 2,524 records with duplicates, 775 duplicates removed, and 1,749 in the deduplicated set. Each row details the progression from initial records, through duplicates removed, to the final deduplicated gold standard set.

Navigate back to Table 2.

### Table 3 Long description

The table has seven columns: Tool, Total records with duplicates, Duplicates removed, Records in deduplicated sets, Removed singulars, Missed duplicates, and Total time in hours and minutes. From top to bottom, the tools and their values are: ASySD with 26,778 total records, 7,248 duplicates removed, 19,530 deduplicated records, 22 removed singulars, 34 missed duplicates, 00:53 time; Covidence with 26,778 records, 6,985 duplicates removed, 19,793 deduplicated, 8 removed singulars, 276 missed duplicates, 00:32; Deduklick with 26,778 records, 7,189 duplicates removed, 19,589 deduplicated, 11 removed singulars, 81 missed duplicates, 00:14; EPPI-Reviewer with 26,778 records, 7,209 duplicates removed, 19,569 deduplicated, 20 removed singulars, 74 missed duplicates, 01:03; PICO Portal with 26,778 records, 7,129 duplicates removed, 19,649 deduplicated, 16 removed singulars, 136 missed duplicates, 05:07; Rayyan with 26,778 records, 7,222 duplicates removed, 19,556 deduplicated, 2 removed singulars, 40 missed duplicates, 20:34; S R A Deduplicator Focused with 26,778 records, 7,191 duplicates removed, 19,587 deduplicated, 9 removed singulars, 81 missed duplicates, 02:58; S R A Deduplicator Relaxed with 26,778 records, 6,976 duplicates removed, 19,802 deduplicated, 11 removed singulars, 280 missed duplicates, 00:01. All tools start with the same number of records but differ in duplicates removed, errors, and processing time.

Navigate back to Table 3.

### Table 4 Long description

From the top row, column headers are Tool, Total records with duplicates (S D): range, Records in deduplicated set (S D): range, Removed singulars (S D), Missed duplicates (S D): range, and Mean time (S D): range minutes. The first row lists ASySD with 5,356 total records (S D 4,084; range 2,524 to 12,432), 3,906 deduplicated (S D 2,207; range 1,744 to 7,302), 4 removed singulars (S D 3; range 0 to 7), 7 missed duplicates (S D 10; range 1 to 25), and mean time 11 minutes (S D 7; range 6 to 23). Covidence has 5,357 total (S D 4,084; range 2,524 to 12,432), 3,959 deduplicated (S D 2,251; range 1,786 to 7,450), 2 removed singulars (S D 1; range 0 to 3), 55 missed duplicates (S D 64; range 21 to 169), mean time 7 minutes (S D 5; range 4 to 14). Deduklick shows 5,358 total (S D 4,084; range 2,524 to 12,432), 3,918 deduplicated (S D 2,217; range 1,753 to 7,333), 2 removed singulars (S D 1; range 1 to 4), 16 missed duplicates (S D 23; range 5 to 57), mean time 3 minutes (S D 1; range 1 to 5). EPPI Reviewer has 5,359 total (S D 4,084; range 2,524 to 12,432), 3,914 deduplicated (S D 2,208; range 1,758 to 7,316), 4 removed singulars (S D 3; range 1 to 8), 15 missed duplicates (S D 17; range 3 to 44), mean time 13 minutes (S D 8; range 3 to 24). PICO Portal lists 5,360 total (S D 4,084; range 2,524 to 12,432), 3,930 deduplicated (S D 2,225; range 1,778 to 7,372), 3 removed singulars (S D 2; range 1 to 6), 27 missed duplicates (S D 36; range 5 to 90), mean time 61 minutes (S D 80; range 11 to 201). Rayyan has 5,361 total (S D 4,084; range 2,524 to 12,432), 3,911 deduplicated (S D 2,205; range 1,755 to 7,305), 0 removed singulars (S D 1; range 0 to 1), 8 missed duplicates (S D 10; range 3 to 25), mean time 247 minutes (S D 292; range 79 to 766). S R A Deduplicator Focused shows 5,363 total (S D 4,084; range 2,524 to 12,432), 3,917 deduplicated (S D 2,211; range 1,753 to 7,323), 2 removed singulars (S D 1; range 0 to 4), 16 missed duplicates (S D 18; range 2 to 45), mean time 36 minutes (S D 27; range 8 to 80). S R A Deduplicator Relaxed lists 5,362 total (S D 4,084; range 2,524 to 12,432), 3,960 deduplicated (S D 2,258; range 1,779 to 7,464), 2 removed singulars (S D 1; range 1 to 4), 56 missed duplicates (S D 68; range 22 to 177), mean time 0.1 minutes (S D 0.1; range 0.08 to 0.32). S D is defined as standard deviation.

Navigate back to Table 4.

### Table 5 Long description

Beginning at the top row, the tools are listed in the first column: Rayyan, Covidence, S R A Deduplicator Focused, Deduklick, S R A Deduplicator Relaxed, PICO Portal, EPPI Reviewer, and ASySD. The second column shows estimates, ranging from minus 9.4049 for Rayyan to minus 7.0060 for ASySD. The third column displays standard errors, from 0.7520 for Rayyan down to 0.3331 for A Sy S D. The fourth column presents mean error rates, bolded, increasing from 0.000082 for Rayyan up to 0.000906 for ASySD. The fifth column shows standard error mean error rates, from 0.000062 for Rayyan to 0.000301 for ASySD. Each row aligns tool performance metrics horizontally, allowing direct comparison across all four quantitative measures.

Navigate back to Table 5.

### Table 6 Long description

Beginning at the top row, the table lists eight tools in the leftmost column: ASySD, Rayyan, EPPI-Reviewer, Deduklick, S R A Deduplicator Focused, PICO Portal, Covidence, and S R A Deduplicator Relaxed. For each tool, the following columns are provided in order: Estimate, Standard error, Mean error rate (bolded), Statistical ranking, and Standard error mean error rate. ASySD has an estimate of minus 6.8921, standard error 0.3063, mean error rate 0.001015, ranking 1, and standard error mean error rate 0.000311. Rayyan shows minus 6.7293, 0.2990, 0.001194, ranking 1, and 0.000357. EPPI-Reviewer has minus 6.1127, 0.2792, 0.002210, ranking 2, and 0.000616. Deduklick and S R A Deduplicator Focused both have minus 6.0220, 0.2771, 0.002419, ranking 2, and 0.000669. PICO Portal displays minus 5.5014, 0.2679, 0.004065, ranking 3, and 0.001084. Covidence has minus 4.7875, 0.2608, 0.008265, ranking 4, and 0.002138. S R A Deduplicator Relaxed shows minus 4.7729, 0.2607, 0.008385, ranking 4, and 0.002168. The lowest mean error rates are observed for ASySD and Rayyan, both ranked first, while Covidence and S R A Deduplicator Relaxed have the highest mean error rates and lowest rankings.

Navigate back to Table 6.

### Table 7 Long description

Beginning at the top row, the columns are Tool, Estimate, Standard error, Mean time in minutes, and Statistical ranking. S R A Deduplicator: Relaxed shows estimate minus 2.1239, standard error 0.3489, mean time 0.11 minutes, ranking 1. Deduklick has estimate 0.9000, standard error 0.3489, mean time 2.4 minutes, ranking 2. Covidence is estimate 1.6809, standard error 0.3489, mean time 5.3 minutes, ranking 3. ASySD is estimate 2.2095, standard error 0.3489, mean time 9.1 minutes, ranking 3. EPPI Reviewer is estimate 2.3406, standard error 0.3489, mean time 10.3 minutes, ranking 3. S R A Deduplicator: Focused is estimate 3.3330, standard error 0.3489, mean time 28.0 minutes, ranking 4. PICO Portal is estimate 3.5127, standard error 0.3489, mean time 33.5 minutes, ranking 4. Rayyan is estimate 5.1022, standard error 0.3489, mean time 164.3 minutes, ranking 5. The fastest tool is S R A Deduplicator: Relaxed, and the slowest is Rayyan.

Navigate back to Table 7.
