# Supplementary material for: Evaluating the accuracy and speed of eight deduplication tools: A comparative study
Source: Res Synth Methods. 2026 Jun 17;17(6):1144–57. doi: 10.1017/rsm.2026.10100 (PMC13732358; doi:10.1017/rsm.2026.10100)
Supplement: Supplementary file 1 [file S1759287926101008sup001.docx]

**The Mixed Procedure**

| **Model Information** | |
| --- | --- |
| **Data Set** | WORK.MYEXCEL2 |
| **Dependent Variable** | logtime |
| **Covariance Structure** | Variance Components |
| **Subject Effect** | Review |
| **Estimation Method** | REML |
| **Residual Variance Method** | Profile |
| **Fixed Effects SE Method** | Model-Based |
| **Degrees of Freedom Method** | Containment |

| **Class Level Information** | | |
| --- | --- | --- |
| **Class** | **Levels** | **Values** |
| **Review** | 5 | Arora Cox Freak Noone Pisano |
| **Method** | 8 | ASySD Covidence Deduklick EPPI_Reviewer PICO_Portal Rayyan SRA_Dedup1 SRA_Dedup2 |

| **Dimensions** | |
| --- | --- |
| **Covariance Parameters** | 2 |
| **Columns in X** | 9 |
| **Columns in Z per Subject** | 1 |
| **Subjects** | 5 |
| **Max Obs per Subject** | 8 |

| **Number of Observations** | |
| --- | --- |
| **Number of Observations Read** | 40 |
| **Number of Observations Used** | 40 |
| **Number of Observations Not Used** | 0 |

| **Iteration History** | | | |
| --- | --- | --- | --- |
| **Iteration** | **Evaluations** | **-2 Res Log Like** | **Criterion** |
| **0** | 1 | 87.80508822 |  |
| **1** | 1 | 71.45069053 | 0.00000000 |

Convergence criteria met.

| **Covariance Parameter Estimates** | | |
| --- | --- | --- |
| **Cov Parm** | **Subject** | **Estimate** |
| **Intercept** | Review | 0.3382 |
| **Residual** |  | 0.2706 |

| **Fit Statistics** | |
| --- | --- |
| **-2 Res Log Likelihood** | 71.5 |
| **AIC (Smaller is Better)** | 75.5 |
| **AICC (Smaller is Better)** | 75.9 |
| **BIC (Smaller is Better)** | 74.7 |

| **Solution for Fixed Effects** | | | | | | |
| --- | --- | --- | --- | --- | --- | --- |
| **Effect** | **Method** | **Estimate** | **Standard Error** | **DF** | **t Value** | **Pr > \|t\|** |
| **Intercept** |  | 3.3330 | 0.3489 | 4 | 9.55 | 0.0007 |
| **Method** | ASySD | -1.1235 | 0.3290 | 28 | -3.41 | 0.0020 |
| **Method** | Covidence | -1.6521 | 0.3290 | 28 | -5.02 | <.0001 |
| **Method** | Deduklick | -2.4331 | 0.3290 | 28 | -7.40 | <.0001 |
| **Method** | EPPI_Reviewer | -0.9924 | 0.3290 | 28 | -3.02 | 0.0054 |
| **Method** | PICO_Portal | 0.1797 | 0.3290 | 28 | 0.55 | 0.5892 |
| **Method** | Rayyan | 1.7692 | 0.3290 | 28 | 5.38 | <.0001 |
| **Method** | SRA_Dedup1 | -5.4569 | 0.3290 | 28 | -16.59 | <.0001 |
| **Method** | SRA_Dedup2 | 0 | . | . | . | . |

| **Type 3 Tests of Fixed Effects** | | | | |
| --- | --- | --- | --- | --- |
| **Effect** | **Num DF** | **Den DF** | **F Value** | **Pr > F** |
| **Method** | 7 | 28 | 84.61 | <.0001 |

| **Least Squares Means** | | | | | | |
| --- | --- | --- | --- | --- | --- | --- |
| **Effect** | **Method** | **Estimate** | **Standard Error** | **DF** | **t Value** | **Pr > \|t\|** |
| **Method** | ASySD | 2.2095 | 0.3489 | 28 | 6.33 | <.0001 |
| **Method** | Covidence | 1.6809 | 0.3489 | 28 | 4.82 | <.0001 |
| **Method** | Deduklick | 0.9000 | 0.3489 | 28 | 2.58 | 0.0154 |
| **Method** | EPPI_Reviewer | 2.3406 | 0.3489 | 28 | 6.71 | <.0001 |
| **Method** | PICO_Portal | 3.5127 | 0.3489 | 28 | 10.07 | <.0001 |
| **Method** | Rayyan | 5.1022 | 0.3489 | 28 | 14.62 | <.0001 |
| **Method** | SRA_Dedup1 | -2.1239 | 0.3489 | 28 | -6.09 | <.0001 |
| **Method** | SRA_Dedup2 | 3.3330 | 0.3489 | 28 | 9.55 | <.0001 |

| **Differences of Least Squares Means** | | | | | | | |
| --- | --- | --- | --- | --- | --- | --- | --- |
| **Effect** | **Method** | **Method** | **Estimate** | **Standard Error** | **DF** | **t Value** | **Pr > \|t\|** |
| **Method** | ASySD | Covidence | 0.5286 | 0.3290 | 28 | 1.61 | 0.1193 |
| **Method** | ASySD | Deduklick | 1.3095 | 0.3290 | 28 | 3.98 | 0.0004 |
| **Method** | ASySD | EPPI_Reviewer | -0.1311 | 0.3290 | 28 | -0.40 | 0.6932 |
| **Method** | ASySD | PICO_Portal | -1.3032 | 0.3290 | 28 | -3.96 | 0.0005 |
| **Method** | ASySD | Rayyan | -2.8927 | 0.3290 | 28 | -8.79 | <.0001 |
| **Method** | ASySD | SRA_Dedup1 | 4.3334 | 0.3290 | 28 | 13.17 | <.0001 |
| **Method** | ASySD | SRA_Dedup2 | -1.1235 | 0.3290 | 28 | -3.41 | 0.0020 |
| **Method** | Covidence | Deduklick | 0.7809 | 0.3290 | 28 | 2.37 | 0.0247 |
| **Method** | Covidence | EPPI_Reviewer | -0.6598 | 0.3290 | 28 | -2.01 | 0.0547 |
| **Method** | Covidence | PICO_Portal | -1.8319 | 0.3290 | 28 | -5.57 | <.0001 |
| **Method** | Covidence | Rayyan | -3.4214 | 0.3290 | 28 | -10.40 | <.0001 |
| **Method** | Covidence | SRA_Dedup1 | 3.8048 | 0.3290 | 28 | 11.56 | <.0001 |
| **Method** | Covidence | SRA_Dedup2 | -1.6521 | 0.3290 | 28 | -5.02 | <.0001 |
| **Method** | Deduklick | EPPI_Reviewer | -1.4407 | 0.3290 | 28 | -4.38 | 0.0002 |
| **Method** | Deduklick | PICO_Portal | -2.6128 | 0.3290 | 28 | -7.94 | <.0001 |
| **Method** | Deduklick | Rayyan | -4.2023 | 0.3290 | 28 | -12.77 | <.0001 |
| **Method** | Deduklick | SRA_Dedup1 | 3.0239 | 0.3290 | 28 | 9.19 | <.0001 |
| **Method** | Deduklick | SRA_Dedup2 | -2.4331 | 0.3290 | 28 | -7.40 | <.0001 |
| **Method** | EPPI_Reviewer | PICO_Portal | -1.1721 | 0.3290 | 28 | -3.56 | 0.0013 |
| **Method** | EPPI_Reviewer | Rayyan | -2.7616 | 0.3290 | 28 | -8.39 | <.0001 |
| **Method** | EPPI_Reviewer | SRA_Dedup1 | 4.4645 | 0.3290 | 28 | 13.57 | <.0001 |
| **Method** | EPPI_Reviewer | SRA_Dedup2 | -0.9924 | 0.3290 | 28 | -3.02 | 0.0054 |
| **Method** | PICO_Portal | Rayyan | -1.5895 | 0.3290 | 28 | -4.83 | <.0001 |
| **Method** | PICO_Portal | SRA_Dedup1 | 5.6367 | 0.3290 | 28 | 17.13 | <.0001 |
| **Method** | PICO_Portal | SRA_Dedup2 | 0.1797 | 0.3290 | 28 | 0.55 | 0.5892 |
| **Method** | Rayyan | SRA_Dedup1 | 7.2261 | 0.3290 | 28 | 21.96 | <.0001 |
| **Method** | Rayyan | SRA_Dedup2 | 1.7692 | 0.3290 | 28 | 5.38 | <.0001 |
| **Method** | SRA_Dedup1 | SRA_Dedup2 | -5.4569 | 0.3290 | 28 | -16.59 | <.0001 |

**The GLIMMIX Procedure**

| **Model Information** | |
| --- | --- |
| **Data Set** | WORK.MYEXCEL2 |
| **Response Variable (Events)** | Missed_Singulars |
| **Response Variable (Trials)** | Total_refs |
| **Response Distribution** | Binomial |
| **Link Function** | Logit |
| **Variance Function** | Default |
| **Variance Matrix Blocked By** | Review |
| **Estimation Technique** | Residual PL |
| **Degrees of Freedom Method** | Containment |

| **Class Level Information** | | |
| --- | --- | --- |
| **Class** | **Levels** | **Values** |
| **Review** | 5 | Arora Cox Freak Noone Pisano |
| **Method** | 8 | ASySD Covidence Deduklick EPPI_Reviewer PICO_Portal Rayyan SRA_Dedup1 SRA_Dedup2 |

| **Number of Observations Read** | 40 |
| --- | --- |
| **Number of Observations Used** | 40 |
| **Number of Events** | 99 |
| **Number of Trials** | 214224 |

| **Dimensions** | |
| --- | --- |
| **G-side Cov. Parameters** | 1 |
| **Columns in X** | 9 |
| **Columns in Z per Subject** | 1 |
| **Subjects (Blocks in V)** | 5 |
| **Max Obs per Subject** | 8 |

| **Optimization Information** | |
| --- | --- |
| **Optimization Technique** | Dual Quasi-Newton |
| **Parameters in Optimization** | 1 |
| **Lower Boundaries** | 1 |
| **Upper Boundaries** | 0 |
| **Fixed Effects** | Profiled |
| **Starting From** | Data |

| **Iteration History** | | | | | |
| --- | --- | --- | --- | --- | --- |
| **Iteration** | **Restarts** | **Subiterations** | **Objective Function** | **Change** | **Max Gradient** |
| **0** | **0** | **3** | 80.800319945 | 0.73149264 | 0.000038 |
| **1** | **0** | **3** | 89.930763166 | 0.07396156 | 0.000018 |
| **2** | **0** | **2** | 91.134225314 | 0.00308464 | 6.94E-7 |
| **3** | **0** | **1** | 91.156706752 | 0.00005174 | 3.499E-8 |
| **4** | **0** | **0** | 91.156762482 | 0.00000000 | 2.312E-6 |

Convergence criterion (PCONV=1.11022E-8) satisfied.

| **Fit Statistics** | |
| --- | --- |
| **-2 Res Log Pseudo-Likelihood** | 91.16 |
| **Generalized Chi-Square** | 31.44 |
| **Gener. Chi-Square / DF** | 0.98 |

| **Covariance Parameter Estimates** | | | |
| --- | --- | --- | --- |
| **Cov Parm** | **Subject** | **Estimate** | **Standard Error** |
| **Intercept** | Review | 0.3190 | 0.2656 |

| **Solutions for Fixed Effects** | | | | | | |
| --- | --- | --- | --- | --- | --- | --- |
| **Effect** | **Method** | **Estimate** | **Standard Error** | **DF** | **t Value** | **Pr > \|t\|** |
| **Intercept** |  | -7.9005 | 0.4202 | 4 | -18.80 | <.0001 |
| **Method** | ASySD | 0.8944 | 0.3958 | 28 | 2.26 | 0.0318 |
| **Method** | Covidence | -0.1178 | 0.4860 | 28 | -0.24 | 0.8102 |
| **Method** | Deduklick | 0.2008 | 0.4496 | 28 | 0.45 | 0.6586 |
| **Method** | EPPI_Reviewer | 0.7990 | 0.4015 | 28 | 1.99 | 0.0564 |
| **Method** | PICO_Portal | 0.5757 | 0.4168 | 28 | 1.38 | 0.1781 |
| **Method** | Rayyan | -1.5044 | 0.7818 | 28 | -1.92 | 0.0645 |
| **Method** | SRA_Dedup1 | 0.2008 | 0.4496 | 28 | 0.45 | 0.6586 |
| **Method** | SRA_Dedup2 | 0 | . | . | . | . |

| **Type III Tests of Fixed Effects** | | | | |
| --- | --- | --- | --- | --- |
| **Effect** | **Num DF** | **Den DF** | **F Value** | **Pr > F** |
| **Method** | 7 | 28 | 2.92 | 0.0201 |

| **Method Least Squares Means** | | | | | | | |
| --- | --- | --- | --- | --- | --- | --- | --- |
| **Method** | **Estimate** | **Standard Error** | **DF** | **t Value** | **Pr > \|t\|** | **Mean** | **Standard Error Mean** |
| ASySD | -7.0060 | 0.3331 | 28 | -21.03 | <.0001 | 0.000906 | 0.000301 |
| Covidence | -8.0183 | 0.4365 | 28 | -18.37 | <.0001 | 0.000329 | 0.000144 |
| Deduklick | -7.6997 | 0.3955 | 28 | -19.47 | <.0001 | 0.000453 | 0.000179 |
| EPPI_Reviewer | -7.1014 | 0.3398 | 28 | -20.90 | <.0001 | 0.000823 | 0.000280 |
| PICO_Portal | -7.3248 | 0.3578 | 28 | -20.47 | <.0001 | 0.000659 | 0.000235 |
| Rayyan | -9.4049 | 0.7520 | 28 | -12.51 | <.0001 | 0.000082 | 0.000062 |
| SRA_Dedup1 | -7.6997 | 0.3955 | 28 | -19.47 | <.0001 | 0.000453 | 0.000179 |
| SRA_Dedup2 | -7.9005 | 0.4202 | 28 | -18.80 | <.0001 | 0.000370 | 0.000156 |

| **Differences of Method Least Squares Means** | | | | | | |
| --- | --- | --- | --- | --- | --- | --- |
| **Method** | **Method** | **Estimate** | **Standard Error** | **DF** | **t Value** | **Pr > \|t\|** |
| ASySD | Covidence | 1.0123 | 0.4130 | 28 | 2.45 | 0.0207 |
| ASySD | Deduklick | 0.6937 | 0.3694 | 28 | 1.88 | 0.0709 |
| ASySD | EPPI_Reviewer | 0.09540 | 0.3091 | 28 | 0.31 | 0.7599 |
| ASySD | PICO_Portal | 0.3187 | 0.3287 | 28 | 0.97 | 0.3405 |
| ASySD | Rayyan | 2.3988 | 0.7386 | 28 | 3.25 | 0.0030 |
| ASySD | SRA_Dedup1 | 0.6937 | 0.3694 | 28 | 1.88 | 0.0709 |
| ASySD | SRA_Dedup2 | 0.8944 | 0.3958 | 28 | 2.26 | 0.0318 |
| Covidence | Deduklick | -0.3186 | 0.4648 | 28 | -0.69 | 0.4987 |
| Covidence | EPPI_Reviewer | -0.9168 | 0.4184 | 28 | -2.19 | 0.0369 |
| Covidence | PICO_Portal | -0.6935 | 0.4331 | 28 | -1.60 | 0.1206 |
| Covidence | Rayyan | 1.3866 | 0.7906 | 28 | 1.75 | 0.0904 |
| Covidence | SRA_Dedup1 | -0.3186 | 0.4648 | 28 | -0.69 | 0.4987 |
| Covidence | SRA_Dedup2 | -0.1178 | 0.4860 | 28 | -0.24 | 0.8102 |
| Deduklick | EPPI_Reviewer | -0.5983 | 0.3755 | 28 | -1.59 | 0.1223 |
| Deduklick | PICO_Portal | -0.3749 | 0.3918 | 28 | -0.96 | 0.3468 |
| Deduklick | Rayyan | 1.7052 | 0.7688 | 28 | 2.22 | 0.0348 |
| Deduklick | SRA_Dedup1 | 1.08E-15 | 0.4265 | 28 | 0.00 | 1.0000 |
| Deduklick | SRA_Dedup2 | 0.2008 | 0.4496 | 28 | 0.45 | 0.6586 |
| EPPI_Reviewer | PICO_Portal | 0.2233 | 0.3355 | 28 | 0.67 | 0.5111 |
| EPPI_Reviewer | Rayyan | 2.3034 | 0.7417 | 28 | 3.11 | 0.0043 |
| EPPI_Reviewer | SRA_Dedup1 | 0.5983 | 0.3755 | 28 | 1.59 | 0.1223 |
| EPPI_Reviewer | SRA_Dedup2 | 0.7990 | 0.4015 | 28 | 1.99 | 0.0564 |
| PICO_Portal | Rayyan | 2.0801 | 0.7501 | 28 | 2.77 | 0.0098 |
| PICO_Portal | SRA_Dedup1 | 0.3749 | 0.3918 | 28 | 0.96 | 0.3468 |
| PICO_Portal | SRA_Dedup2 | 0.5757 | 0.4168 | 28 | 1.38 | 0.1781 |
| Rayyan | SRA_Dedup1 | -1.7052 | 0.7688 | 28 | -2.22 | 0.0348 |
| Rayyan | SRA_Dedup2 | -1.5044 | 0.7818 | 28 | -1.92 | 0.0645 |
| SRA_Dedup1 | SRA_Dedup2 | 0.2008 | 0.4496 | 28 | 0.45 | 0.6586 |

**The GLIMMIX Procedure**

| **Model Information** | |
| --- | --- |
| **Data Set** | WORK.MYEXCEL2 |
| **Response Variable (Events)** | Missed_Dups |
| **Response Variable (Trials)** | Total_refs |
| **Response Distribution** | Binomial |
| **Link Function** | Logit |
| **Variance Function** | Default |
| **Variance Matrix Blocked By** | Review |
| **Estimation Technique** | Residual PL |
| **Degrees of Freedom Method** | Containment |

| **Class Level Information** | | |
| --- | --- | --- |
| **Class** | **Levels** | **Values** |
| **Review** | 5 | Arora Cox Freak Noone Pisano |
| **Method** | 8 | ASySD Covidence Deduklick EPPI_Reviewer PICO_Portal Rayyan SRA_Dedup1 SRA_Dedup2 |

| **Number of Observations Read** | 40 |
| --- | --- |
| **Number of Observations Used** | 40 |
| **Number of Events** | 1002 |
| **Number of Trials** | 214224 |

| **Dimensions** | |
| --- | --- |
| **G-side Cov. Parameters** | 1 |
| **Columns in X** | 9 |
| **Columns in Z per Subject** | 1 |
| **Subjects (Blocks in V)** | 5 |
| **Max Obs per Subject** | 8 |

| **Optimization Information** | |
| --- | --- |
| **Optimization Technique** | Dual Quasi-Newton |
| **Parameters in Optimization** | 1 |
| **Lower Boundaries** | 1 |
| **Upper Boundaries** | 0 |
| **Fixed Effects** | Profiled |
| **Starting From** | Data |

| **Iteration History** | | | | | |
| --- | --- | --- | --- | --- | --- |
| **Iteration** | **Restarts** | **Subiterations** | **Objective Function** | **Change** | **Max Gradient** |
| **0** | **0** | **3** | 64.308149935 | 1.70827707 | 0.000014 |
| **1** | **0** | **2** | 73.143141642 | 1.97843054 | 5.706E-7 |
| **2** | **0** | **1** | 73.6915175 | 1.99989397 | 2.152E-7 |
| **3** | **0** | **0** | 73.693720684 | 1.99998610 | 2.705E-6 |
| **4** | **0** | **0** | 73.693720733 | 0.00000000 | 2.705E-6 |

Convergence criterion (PCONV=1.11022E-8) satisfied.

| **Fit Statistics** | |
| --- | --- |
| **-2 Res Log Pseudo-Likelihood** | 73.69 |
| **Generalized Chi-Square** | 65.40 |
| **Gener. Chi-Square / DF** | 2.04 |

| **Covariance Parameter Estimates** | | | |
| --- | --- | --- | --- |
| **Cov Parm** | **Subject** | **Estimate** | **Standard Error** |
| **Intercept** | Review | 0.3176 | 0.2320 |

| **Solutions for Fixed Effects** | | | | | | |
| --- | --- | --- | --- | --- | --- | --- |
| **Effect** | **Method** | **Estimate** | **Standard Error** | **DF** | **t Value** | **Pr > \|t\|** |
| **Intercept** |  | -6.0220 | 0.2771 | 4 | -21.73 | <.0001 |
| **Method** | ASySD | -0.8701 | 0.2046 | 28 | -4.25 | 0.0002 |
| **Method** | Covidence | 1.2345 | 0.1267 | 28 | 9.74 | <.0001 |
| **Method** | Deduklick | -222E-19 | 0.1574 | 28 | -0.00 | 1.0000 |
| **Method** | EPPI_Reviewer | -0.09069 | 0.1611 | 28 | -0.56 | 0.5779 |
| **Method** | PICO_Portal | 0.5206 | 0.1407 | 28 | 3.70 | 0.0009 |
| **Method** | Rayyan | -0.7074 | 0.1935 | 28 | -3.66 | 0.0010 |
| **Method** | SRA_Dedup1 | 1.2491 | 0.1265 | 28 | 9.87 | <.0001 |
| **Method** | SRA_Dedup2 | 0 | . | . | . | . |

| **Type III Tests of Fixed Effects** | | | | |
| --- | --- | --- | --- | --- |
| **Effect** | **Num DF** | **Den DF** | **F Value** | **Pr > F** |
| **Method** | 7 | 28 | 64.80 | <.0001 |

| **Method Least Squares Means** | | | | | | | |
| --- | --- | --- | --- | --- | --- | --- | --- |
| **Method** | **Estimate** | **Standard Error** | **DF** | **t Value** | **Pr > \|t\|** | **Mean** | **Standard Error Mean** |
| ASySD | -6.8921 | 0.3063 | 28 | -22.50 | <.0001 | 0.001015 | 0.000311 |
| Covidence | -4.7875 | 0.2608 | 28 | -18.35 | <.0001 | 0.008265 | 0.002138 |
| Deduklick | -6.0220 | 0.2771 | 28 | -21.73 | <.0001 | 0.002419 | 0.000669 |
| EPPI_Reviewer | -6.1127 | 0.2792 | 28 | -21.90 | <.0001 | 0.002210 | 0.000616 |
| PICO_Portal | -5.5014 | 0.2679 | 28 | -20.53 | <.0001 | 0.004065 | 0.001084 |
| Rayyan | -6.7293 | 0.2990 | 28 | -22.50 | <.0001 | 0.001194 | 0.000357 |
| SRA_Dedup1 | -4.7729 | 0.2607 | 28 | -18.31 | <.0001 | 0.008385 | 0.002168 |
| SRA_Dedup2 | -6.0220 | 0.2771 | 28 | -21.73 | <.0001 | 0.002419 | 0.000669 |

| **Differences of Method Least Squares Means** | | | | | | |
| --- | --- | --- | --- | --- | --- | --- |
| **Method** | **Method** | **Estimate** | **Standard Error** | **DF** | **t Value** | **Pr > \|t\|** |
| ASySD | Covidence | -2.1047 | 0.1820 | 28 | -11.56 | <.0001 |
| ASySD | Deduklick | -0.8701 | 0.2046 | 28 | -4.25 | 0.0002 |
| ASySD | EPPI_Reviewer | -0.7795 | 0.2074 | 28 | -3.76 | 0.0008 |
| ASySD | PICO_Portal | -1.3908 | 0.1920 | 28 | -7.24 | <.0001 |
| ASySD | Rayyan | -0.1628 | 0.2335 | 28 | -0.70 | 0.4914 |
| ASySD | SRA_Dedup1 | -2.1192 | 0.1819 | 28 | -11.65 | <.0001 |
| ASySD | SRA_Dedup2 | -0.8701 | 0.2046 | 28 | -4.25 | 0.0002 |
| Covidence | Deduklick | 1.2345 | 0.1267 | 28 | 9.74 | <.0001 |
| Covidence | EPPI_Reviewer | 1.3252 | 0.1312 | 28 | 10.10 | <.0001 |
| Covidence | PICO_Portal | 0.7139 | 0.1052 | 28 | 6.79 | <.0001 |
| Covidence | Rayyan | 1.9419 | 0.1694 | 28 | 11.46 | <.0001 |
| Covidence | SRA_Dedup1 | -0.01456 | 0.08534 | 28 | -0.17 | 0.8657 |
| Covidence | SRA_Dedup2 | 1.2345 | 0.1267 | 28 | 9.74 | <.0001 |
| Deduklick | EPPI_Reviewer | 0.09069 | 0.1611 | 28 | 0.56 | 0.5779 |
| Deduklick | PICO_Portal | -0.5206 | 0.1407 | 28 | -3.70 | 0.0009 |
| Deduklick | Rayyan | 0.7074 | 0.1935 | 28 | 3.66 | 0.0010 |
| Deduklick | SRA_Dedup1 | -1.2491 | 0.1265 | 28 | -9.87 | <.0001 |
| Deduklick | SRA_Dedup2 | -222E-19 | 0.1574 | 28 | -0.00 | 1.0000 |
| EPPI_Reviewer | PICO_Portal | -0.6113 | 0.1448 | 28 | -4.22 | 0.0002 |
| EPPI_Reviewer | Rayyan | 0.6167 | 0.1965 | 28 | 3.14 | 0.0040 |
| EPPI_Reviewer | SRA_Dedup1 | -1.3398 | 0.1310 | 28 | -10.22 | <.0001 |
| EPPI_Reviewer | SRA_Dedup2 | -0.09069 | 0.1611 | 28 | -0.56 | 0.5779 |
| PICO_Portal | Rayyan | 1.2280 | 0.1801 | 28 | 6.82 | <.0001 |
| PICO_Portal | SRA_Dedup1 | -0.7285 | 0.1049 | 28 | -6.94 | <.0001 |
| PICO_Portal | SRA_Dedup2 | 0.5206 | 0.1407 | 28 | 3.70 | 0.0009 |
| Rayyan | SRA_Dedup1 | -1.9564 | 0.1693 | 28 | -11.56 | <.0001 |
| Rayyan | SRA_Dedup2 | -0.7074 | 0.1935 | 28 | -3.66 | 0.0010 |
| SRA_Dedup1 | SRA_Dedup2 | 1.2491 | 0.1265 | 28 | 9.87 | <.0001 |

**The GLIMMIX Procedure**

| **Model Information** | |
| --- | --- |
| **Data Set** | WORK.MYEXCEL2 |
| **Response Variable** | Missed_Singulars |
| **Response Distribution** | Poisson |
| **Link Function** | Log |
| **Variance Function** | Default |
| **Offset Variable** | off |
| **Variance Matrix Blocked By** | Review |
| **Estimation Technique** | Residual PL |
| **Degrees of Freedom Method** | Containment |

| **Class Level Information** | | |
| --- | --- | --- |
| **Class** | **Levels** | **Values** |
| **Review** | 5 | Arora Cox Freak Noone Pisano |
| **Method** | 8 | ASySD Covidence Deduklick EPPI_Reviewer PICO_Portal Rayyan SRA_Dedup1 SRA_Dedup2 |

| **Number of Observations Read** | 40 |
| --- | --- |
| **Number of Observations Used** | 40 |

| **Dimensions** | |
| --- | --- |
| **G-side Cov. Parameters** | 1 |
| **Columns in X** | 9 |
| **Columns in Z per Subject** | 1 |
| **Subjects (Blocks in V)** | 5 |
| **Max Obs per Subject** | 8 |

| **Optimization Information** | |
| --- | --- |
| **Optimization Technique** | Dual Quasi-Newton |
| **Parameters in Optimization** | 1 |
| **Lower Boundaries** | 1 |
| **Upper Boundaries** | 0 |
| **Fixed Effects** | Profiled |
| **Starting From** | Data |

| **Iteration History** | | | | | |
| --- | --- | --- | --- | --- | --- |
| **Iteration** | **Restarts** | **Subiterations** | **Objective Function** | **Change** | **Max Gradient** |
| **0** | **0** | **3** | 80.754068428 | 0.73204154 | 0.000038 |
| **1** | **0** | **3** | 89.893428885 | 0.07416122 | 0.000018 |
| **2** | **0** | **2** | 91.099257462 | 0.00309268 | 7.044E-7 |
| **3** | **0** | **1** | 91.121812134 | 0.00005194 | 4.167E-8 |
| **4** | **0** | **0** | 91.121868059 | 0.00000000 | 2.32E-6 |

Convergence criterion (PCONV=1.11022E-8) satisfied.

| **Fit Statistics** | |
| --- | --- |
| **-2 Res Log Pseudo-Likelihood** | 91.12 |
| **Generalized Chi-Square** | 31.42 |
| **Gener. Chi-Square / DF** | 0.98 |

| **Covariance Parameter Estimates** | | | |
| --- | --- | --- | --- |
| **Cov Parm** | **Subject** | **Estimate** | **Standard Error** |
| **Intercept** | Review | 0.3185 | 0.2652 |

| **Solutions for Fixed Effects** | | | | | | |
| --- | --- | --- | --- | --- | --- | --- |
| **Effect** | **Method** | **Estimate** | **Standard Error** | **DF** | **t Value** | **Pr > \|t\|** |
| **Intercept** |  | -7.9009 | 0.4201 | 4 | -18.81 | <.0001 |
| **Method** | ASySD | 0.8938 | 0.3957 | 28 | 2.26 | 0.0319 |
| **Method** | Covidence | -0.1178 | 0.4859 | 28 | -0.24 | 0.8102 |
| **Method** | Deduklick | 0.2007 | 0.4495 | 28 | 0.45 | 0.6587 |
| **Method** | EPPI_Reviewer | 0.7985 | 0.4014 | 28 | 1.99 | 0.0565 |
| **Method** | PICO_Portal | 0.5754 | 0.4167 | 28 | 1.38 | 0.1782 |
| **Method** | Rayyan | -1.5041 | 0.7817 | 28 | -1.92 | 0.0646 |
| **Method** | SRA_Dedup1 | 0.2007 | 0.4495 | 28 | 0.45 | 0.6587 |
| **Method** | SRA_Dedup2 | 0 | . | . | . | . |

| **Type III Tests of Fixed Effects** | | | | |
| --- | --- | --- | --- | --- |
| **Effect** | **Num DF** | **Den DF** | **F Value** | **Pr > F** |
| **Method** | 7 | 28 | 2.92 | 0.0201 |

| **Method Least Squares Means** | | | | | | | |
| --- | --- | --- | --- | --- | --- | --- | --- |
| **Method** | **Estimate** | **Standard Error** | **DF** | **t Value** | **Pr > \|t\|** | **Mean** | **Standard Error Mean** |
| ASySD | -7.0070 | 0.3329 | 28 | -21.05 | <.0001 | 0.000905 | 0.000301 |
| Covidence | -8.0186 | 0.4363 | 28 | -18.38 | <.0001 | 0.000329 | 0.000144 |
| Deduklick | -7.7002 | 0.3953 | 28 | -19.48 | <.0001 | 0.000453 | 0.000179 |
| EPPI_Reviewer | -7.1023 | 0.3396 | 28 | -20.91 | <.0001 | 0.000823 | 0.000280 |
| PICO_Portal | -7.3255 | 0.3576 | 28 | -20.49 | <.0001 | 0.000659 | 0.000235 |
| Rayyan | -9.4049 | 0.7519 | 28 | -12.51 | <.0001 | 0.000082 | 0.000062 |
| SRA_Dedup1 | -7.7002 | 0.3953 | 28 | -19.48 | <.0001 | 0.000453 | 0.000179 |
| SRA_Dedup2 | -7.9009 | 0.4201 | 28 | -18.81 | <.0001 | 0.000370 | 0.000156 |

| **Differences of Method Least Squares Means** | | | | | | |
| --- | --- | --- | --- | --- | --- | --- |
| **Method** | **Method** | **Estimate** | **Standard Error** | **DF** | **t Value** | **Pr > \|t\|** |
| ASySD | Covidence | 1.0116 | 0.4129 | 28 | 2.45 | 0.0208 |
| ASySD | Deduklick | 0.6931 | 0.3693 | 28 | 1.88 | 0.0710 |
| ASySD | EPPI_Reviewer | 0.09531 | 0.3090 | 28 | 0.31 | 0.7600 |
| ASySD | PICO_Portal | 0.3185 | 0.3286 | 28 | 0.97 | 0.3407 |
| ASySD | Rayyan | 2.3979 | 0.7385 | 28 | 3.25 | 0.0030 |
| ASySD | SRA_Dedup1 | 0.6931 | 0.3693 | 28 | 1.88 | 0.0710 |
| ASySD | SRA_Dedup2 | 0.8938 | 0.3957 | 28 | 2.26 | 0.0319 |
| Covidence | Deduklick | -0.3185 | 0.4647 | 28 | -0.69 | 0.4988 |
| Covidence | EPPI_Reviewer | -0.9163 | 0.4183 | 28 | -2.19 | 0.0370 |
| Covidence | PICO_Portal | -0.6931 | 0.4330 | 28 | -1.60 | 0.1207 |
| Covidence | Rayyan | 1.3863 | 0.7906 | 28 | 1.75 | 0.0905 |
| Covidence | SRA_Dedup1 | -0.3185 | 0.4647 | 28 | -0.69 | 0.4988 |
| Covidence | SRA_Dedup2 | -0.1178 | 0.4859 | 28 | -0.24 | 0.8102 |
| Deduklick | EPPI_Reviewer | -0.5978 | 0.3754 | 28 | -1.59 | 0.1225 |
| Deduklick | PICO_Portal | -0.3747 | 0.3917 | 28 | -0.96 | 0.3469 |
| Deduklick | Rayyan | 1.7047 | 0.7687 | 28 | 2.22 | 0.0349 |
| Deduklick | SRA_Dedup1 | -5E-16 | 0.4264 | 28 | -0.00 | 1.0000 |
| Deduklick | SRA_Dedup2 | 0.2007 | 0.4495 | 28 | 0.45 | 0.6587 |
| EPPI_Reviewer | PICO_Portal | 0.2231 | 0.3354 | 28 | 0.67 | 0.5113 |
| EPPI_Reviewer | Rayyan | 2.3026 | 0.7416 | 28 | 3.10 | 0.0043 |
| EPPI_Reviewer | SRA_Dedup1 | 0.5978 | 0.3754 | 28 | 1.59 | 0.1225 |
| EPPI_Reviewer | SRA_Dedup2 | 0.7985 | 0.4014 | 28 | 1.99 | 0.0565 |
| PICO_Portal | Rayyan | 2.0794 | 0.7500 | 28 | 2.77 | 0.0098 |
| PICO_Portal | SRA_Dedup1 | 0.3747 | 0.3917 | 28 | 0.96 | 0.3469 |
| PICO_Portal | SRA_Dedup2 | 0.5754 | 0.4167 | 28 | 1.38 | 0.1782 |
| Rayyan | SRA_Dedup1 | -1.7047 | 0.7687 | 28 | -2.22 | 0.0349 |
| Rayyan | SRA_Dedup2 | -1.5041 | 0.7817 | 28 | -1.92 | 0.0646 |
| SRA_Dedup1 | SRA_Dedup2 | 0.2007 | 0.4495 | 28 | 0.45 | 0.6587 |

**The GLIMMIX Procedure**

| **Model Information** | |
| --- | --- |
| **Data Set** | WORK.MYEXCEL2 |
| **Response Variable** | Missed_Dups |
| **Response Distribution** | Poisson |
| **Link Function** | Log |
| **Variance Function** | Default |
| **Offset Variable** | off |
| **Variance Matrix Blocked By** | Review |
| **Estimation Technique** | Residual PL |
| **Degrees of Freedom Method** | Containment |

| **Class Level Information** | | |
| --- | --- | --- |
| **Class** | **Levels** | **Values** |
| **Review** | 5 | Arora Cox Freak Noone Pisano |
| **Method** | 8 | ASySD Covidence Deduklick EPPI_Reviewer PICO_Portal Rayyan SRA_Dedup1 SRA_Dedup2 |

| **Number of Observations Read** | 40 |
| --- | --- |
| **Number of Observations Used** | 40 |

| **Dimensions** | |
| --- | --- |
| **G-side Cov. Parameters** | 1 |
| **Columns in X** | 9 |
| **Columns in Z per Subject** | 1 |
| **Subjects (Blocks in V)** | 5 |
| **Max Obs per Subject** | 8 |

| **Optimization Information** | |
| --- | --- |
| **Optimization Technique** | Dual Quasi-Newton |
| **Parameters in Optimization** | 1 |
| **Lower Boundaries** | 1 |
| **Upper Boundaries** | 0 |
| **Fixed Effects** | Profiled |
| **Starting From** | Data |

| **Iteration History** | | | | | |
| --- | --- | --- | --- | --- | --- |
| **Iteration** | **Restarts** | **Subiterations** | **Objective Function** | **Change** | **Max Gradient** |
| **0** | **0** | **3** | 63.893240856 | 1.70651431 | 0.000015 |
| **1** | **0** | **2** | 72.750974906 | 1.97816801 | 6.472E-7 |
| **2** | **0** | **1** | 73.303918065 | 1.99989132 | 2.367E-7 |
| **3** | **0** | **0** | 73.30616569 | 1.99999435 | 2.814E-6 |
| **4** | **0** | **0** | 73.306165741 | 0.00000000 | 2.814E-6 |

Convergence criterion (PCONV=1.11022E-8) satisfied.

| **Fit Statistics** | |
| --- | --- |
| **-2 Res Log Pseudo-Likelihood** | 73.31 |
| **Generalized Chi-Square** | 65.19 |
| **Gener. Chi-Square / DF** | 2.04 |

| **Covariance Parameter Estimates** | | | |
| --- | --- | --- | --- |
| **Cov Parm** | **Subject** | **Estimate** | **Standard Error** |
| **Intercept** | Review | 0.3141 | 0.2295 |

| **Solutions for Fixed Effects** | | | | | | |
| --- | --- | --- | --- | --- | --- | --- |
| **Effect** | **Method** | **Estimate** | **Standard Error** | **DF** | **t Value** | **Pr > \|t\|** |
| **Intercept** |  | -6.0235 | 0.2757 | 4 | -21.85 | <.0001 |
| **Method** | ASySD | -0.8681 | 0.2043 | 28 | -4.25 | 0.0002 |
| **Method** | Covidence | 1.2260 | 0.1264 | 28 | 9.70 | <.0001 |
| **Method** | Deduklick | 8.88E-16 | 0.1571 | 28 | 0.00 | 1.0000 |
| **Method** | EPPI_Reviewer | -0.09038 | 0.1608 | 28 | -0.56 | 0.5785 |
| **Method** | PICO_Portal | 0.5182 | 0.1404 | 28 | 3.69 | 0.0010 |
| **Method** | Rayyan | -0.7056 | 0.1933 | 28 | -3.65 | 0.0011 |
| **Method** | SRA_Dedup1 | 1.2403 | 0.1262 | 28 | 9.83 | <.0001 |
| **Method** | SRA_Dedup2 | 0 | . | . | . | . |

| **Type III Tests of Fixed Effects** | | | | |
| --- | --- | --- | --- | --- |
| **Effect** | **Num DF** | **Den DF** | **F Value** | **Pr > F** |
| **Method** | 7 | 28 | 64.39 | <.0001 |

| **Method Least Squares Means** | | | | | | | |
| --- | --- | --- | --- | --- | --- | --- | --- |
| **Method** | **Estimate** | **Standard Error** | **DF** | **t Value** | **Pr > \|t\|** | **Mean** | **Standard Error Mean** |
| ASySD | -6.8916 | 0.3051 | 28 | -22.59 | <.0001 | 0.001016 | 0.000310 |
| Covidence | -4.7975 | 0.2594 | 28 | -18.49 | <.0001 | 0.008250 | 0.002140 |
| Deduklick | -6.0235 | 0.2757 | 28 | -21.85 | <.0001 | 0.002421 | 0.000668 |
| EPPI_Reviewer | -6.1139 | 0.2778 | 28 | -22.01 | <.0001 | 0.002212 | 0.000615 |
| PICO_Portal | -5.5053 | 0.2665 | 28 | -20.66 | <.0001 | 0.004065 | 0.001083 |
| Rayyan | -6.7291 | 0.2978 | 28 | -22.60 | <.0001 | 0.001196 | 0.000356 |
| SRA_Dedup1 | -4.7832 | 0.2593 | 28 | -18.45 | <.0001 | 0.008370 | 0.002170 |
| SRA_Dedup2 | -6.0235 | 0.2757 | 28 | -21.85 | <.0001 | 0.002421 | 0.000668 |

| **Differences of Method Least Squares Means** | | | | | | |
| --- | --- | --- | --- | --- | --- | --- |
| **Method** | **Method** | **Estimate** | **Standard Error** | **DF** | **t Value** | **Pr > \|t\|** |
| ASySD | Covidence | -2.0940 | 0.1818 | 28 | -11.52 | <.0001 |
| ASySD | Deduklick | -0.8681 | 0.2043 | 28 | -4.25 | 0.0002 |
| ASySD | EPPI_Reviewer | -0.7777 | 0.2072 | 28 | -3.75 | 0.0008 |
| ASySD | PICO_Portal | -1.3863 | 0.1917 | 28 | -7.23 | <.0001 |
| ASySD | Rayyan | -0.1625 | 0.2333 | 28 | -0.70 | 0.4917 |
| ASySD | SRA_Dedup1 | -2.1084 | 0.1816 | 28 | -11.61 | <.0001 |
| ASySD | SRA_Dedup2 | -0.8681 | 0.2043 | 28 | -4.25 | 0.0002 |
| Covidence | Deduklick | 1.2260 | 0.1264 | 28 | 9.70 | <.0001 |
| Covidence | EPPI_Reviewer | 1.3163 | 0.1309 | 28 | 10.06 | <.0001 |
| Covidence | PICO_Portal | 0.7077 | 0.1048 | 28 | 6.76 | <.0001 |
| Covidence | Rayyan | 1.9315 | 0.1692 | 28 | 11.42 | <.0001 |
| Covidence | SRA_Dedup1 | -0.01439 | 0.08482 | 28 | -0.17 | 0.8665 |
| Covidence | SRA_Dedup2 | 1.2260 | 0.1264 | 28 | 9.70 | <.0001 |
| Deduklick | EPPI_Reviewer | 0.09038 | 0.1608 | 28 | 0.56 | 0.5785 |
| Deduklick | PICO_Portal | -0.5182 | 0.1404 | 28 | -3.69 | 0.0010 |
| Deduklick | Rayyan | 0.7056 | 0.1933 | 28 | 3.65 | 0.0011 |
| Deduklick | SRA_Dedup1 | -1.2403 | 0.1262 | 28 | -9.83 | <.0001 |
| Deduklick | SRA_Dedup2 | 8.88E-16 | 0.1571 | 28 | 0.00 | 1.0000 |
| EPPI_Reviewer | PICO_Portal | -0.6086 | 0.1445 | 28 | -4.21 | 0.0002 |
| EPPI_Reviewer | Rayyan | 0.6152 | 0.1962 | 28 | 3.13 | 0.0040 |
| EPPI_Reviewer | SRA_Dedup1 | -1.3307 | 0.1307 | 28 | -10.18 | <.0001 |
| EPPI_Reviewer | SRA_Dedup2 | -0.09038 | 0.1608 | 28 | -0.56 | 0.5785 |
| PICO_Portal | Rayyan | 1.2238 | 0.1799 | 28 | 6.80 | <.0001 |
| PICO_Portal | SRA_Dedup1 | -0.7221 | 0.1045 | 28 | -6.91 | <.0001 |
| PICO_Portal | SRA_Dedup2 | 0.5182 | 0.1404 | 28 | 3.69 | 0.0010 |
| Rayyan | SRA_Dedup1 | -1.9459 | 0.1690 | 28 | -11.51 | <.0001 |
| Rayyan | SRA_Dedup2 | -0.7056 | 0.1933 | 28 | -3.65 | 0.0011 |
| SRA_Dedup1 | SRA_Dedup2 | 1.2403 | 0.1262 | 28 | 9.83 | <.0001 |
